# Analysis of food system drivers of deforestation highlights foreign direct investments and urbanization as threats to tropical forests

**DOI:** 10.1038/s41598-024-65397-3

**Published:** 2024-07-16

**Authors:** Janelle M. Sylvester, Diana María Gutiérrez-Zapata, Lisset Pérez-Marulanda, Martha Vanegas-Cubillos, Thilde Bech Bruun, Ole Mertz, Augusto Castro-Nunez

**Affiliations:** 1 International Center for Tropical Agriculture (CIAT), Km 17 Recta Cali-Palmira, Cali, Colombia; 2 Department of Geosciences and Natural Resource Management, University of Copenhagen, Copenhagen, Denmark

**Keywords:** Climate-change mitigation, Biodiversity, Ecosystem services, Forestry, Environmental sciences

## Abstract

Approximately 90% of global forest cover changes between 2000 and 2018 were attributable to agricultural expansion, making food production the leading direct driver of deforestation. While previous studies have focused on the interaction between human and environmental systems, limited research has explored deforestation from a food system perspective. This study analyzes the drivers of deforestation in 40 tropical and subtropical countries (2004–2021) through the lenses of consumption/demand, production/supply and trade/distribution using Extreme Gradient Boosting (XGBoost) models. Our models explained a substantial portion of deforestation variability globally (R^2^ = 0.74) and in Asia (R^2^ = 0.81) and Latin America (R^2^ = 0.73). The results indicate that trade- and demand-side dynamics, specifically foreign direct investments and urban population growth, play key roles in influencing deforestation trends at these scales, suggesting that food system-based interventions could be effective in mitigating deforestation. Conversely, the model for Africa showed weaker explanatory power (R^2^ = 0.30), suggesting that factors beyond the food system may play a larger role in this region. Our findings highlight the importance of targeting trade- and demand-side dynamics to reduce deforestation and how interventions within the food system could synergistically contribute to achieving sustainable development goals, such as climate action, life on land and zero hunger.

## Introduction

Agriculture plays an indisputable role in driving forest cover change, associated greenhouse gas (GHG) emissions and biodiversity loss. Approximately 90% of global deforestation between 2000 and 2019 was attributable to crop and pastureland expansion, positing commodity production as the primary direct driver of deforestation^1^. This interaction between land use and land cover change has prompted a breadth of research arising from land systems science that has shed light on the complex drivers of forest cover loss^2–4^. Yet, despite the disproportionate role of food production in driving deforestation, limited research has examined deforestation as an outcome of the food system.

The food system is comprised of all the activities and elements, including environment, people and institutions, involved in every facet of food production and consumption. Food system outcomes, which are the results or consequences of activities within the food system, are typically associated with food and nutrition security (e.g. malnutrition, access to food), socioeconomic (e.g. poverty reduction, income generation) and environmental (e.g. GHG emissions, forest cover and biodiversity loss) outputs^5–9^. Deforestation as an outcome of the food system can occur directly (i.e. conversion of forest to agriculture) or indirectly (i.e. increased demand of forest-risk commodities). The latter underscores the emerging understanding that shifts in food system dynamics in one region can exert far-reaching influences on patterns of land use change in distant regions. Growing recognition of such telecouplings^10–12^ has placed a spotlight on the role of globalization, notably through expanding urbanization, increasing wealth and international trade, in shaping contemporary consumption patterns and land use dynamics^13–15^.

Increasing empirical evidence linking international trade to deforestation and associated GHG emissions^16,17^ has led to a wave of efforts by public and private actors to eliminate deforestation from supply chains^18–20^. While these initiatives, primarily targeting commodity production for export markets, have had success with specific commodities in certain regions, their overall impact on the global deforestation rate has been limited, partly due to growing domestic consumption of commodities in tropical regions^20^. Alongside international trade, associations between urban population growth and deforestation have suggested that both urban-based and international demands for agricultural products serve as critical drivers of deforestation^16,17,21–23^.

Underpinning the links between international trade, growing urbanization and deforestation are changes in consumption and consequential demand for agricultural products, which as a whole transpire against a backdrop of complex national and transnational socioeconomic trends associated with globalization. Consumption changes associated with urbanization are manifested in a growing demand for animal-based products and processed foods driven by a complex of factors that in part include, respectively, increasing incomes and the convenience (in terms of time and labor) of processed foods as family members (particularly women) in urban areas increasingly join the workforce outside the home^15,24–28^. While these consumption-demand dynamics have undeniably contributed to changes in food systems in developing countries, trade-distribution dynamics, such as trade liberalization and internationalization of private investments, have facilitated the supermarket-based distribution of these foods in parallel with growing demands, likewise contributing to food system transformations in these regions^24,29,30^. Trade policies and foreign and national investments have also driven changes in production-supply dynamics through, for instance, increasing access of small and medium producers to domestic and international markets and investments in the production of particular commodities^31,32^. For instance, foreign direct investments (FDI) in agriculture have been linked to the area expansion of crops commonly used in processed foods (e.g. soy, oil palm and sugarcane) in the Global South^33^. These processes not only affect the food system itself but the environmental outcomes it generates.

Béné et al.^24^ illustrates how consumption-demand, production-supply and trade-distribution dynamics drive global food system changes. These food system dimensions offer a structured lens through which we can better comprehend and disentangle the forces that drive deforestation as an outcome of the food system. Consumption-demand related drivers encompass dynamics that drive changes in the human diet and subsequent demand for food, such as urbanization being linked with increased demand for processed foods^26,27^ and a rise in consumers’ income (in low- and middle-income countries) being associated with growing demand for animal-based protein^24,34,35^. Production-supply related drivers concern dynamics that drive changes in agricultural yields and productivity, such as (1) agricultural technological innovation and intensification and homogenization of the agricultural sector (which can increase productivity^36^) and (2) climate change and degradation of agroecosystems (which can drive productivity declines^37,38^). This category also considers dynamics affecting the supply of food, such as improved access to local infrastructure, supermarkets and growing urban centers^24^. Trade-distribution related drivers encompass dynamics related to the globalization of food trade, which is influenced by factors such as the internationalization of private investments^39^ and growing attention to and concerns over food safety^24^.

This study is one of the first attempts to comprehensively model food system dynamics as drivers of deforestation. In doing so, we present an innovative analytical approach structured around the food system’s dimensions to understand how drivers related to consumption-demand, production-supply and trade-distribution drive deforestation. We employ a machine learning approach utilizing the Extreme Gradient Boosting (XGBoost) algorithm and data from 40 countries spanning 2004–2021 to construct models at global and regional scales. Our findings suggest that cross-sectoral FDI and growing urban populations are critical drivers of environmental outcomes of the food system. We dissect the role these variables have played in transforming food systems and the possible implications this has had for deforestation. Lastly, we provide insights that can inform policy strategies for mitigating deforestation while promoting sustainable development.

## Results

**Figure 1:**
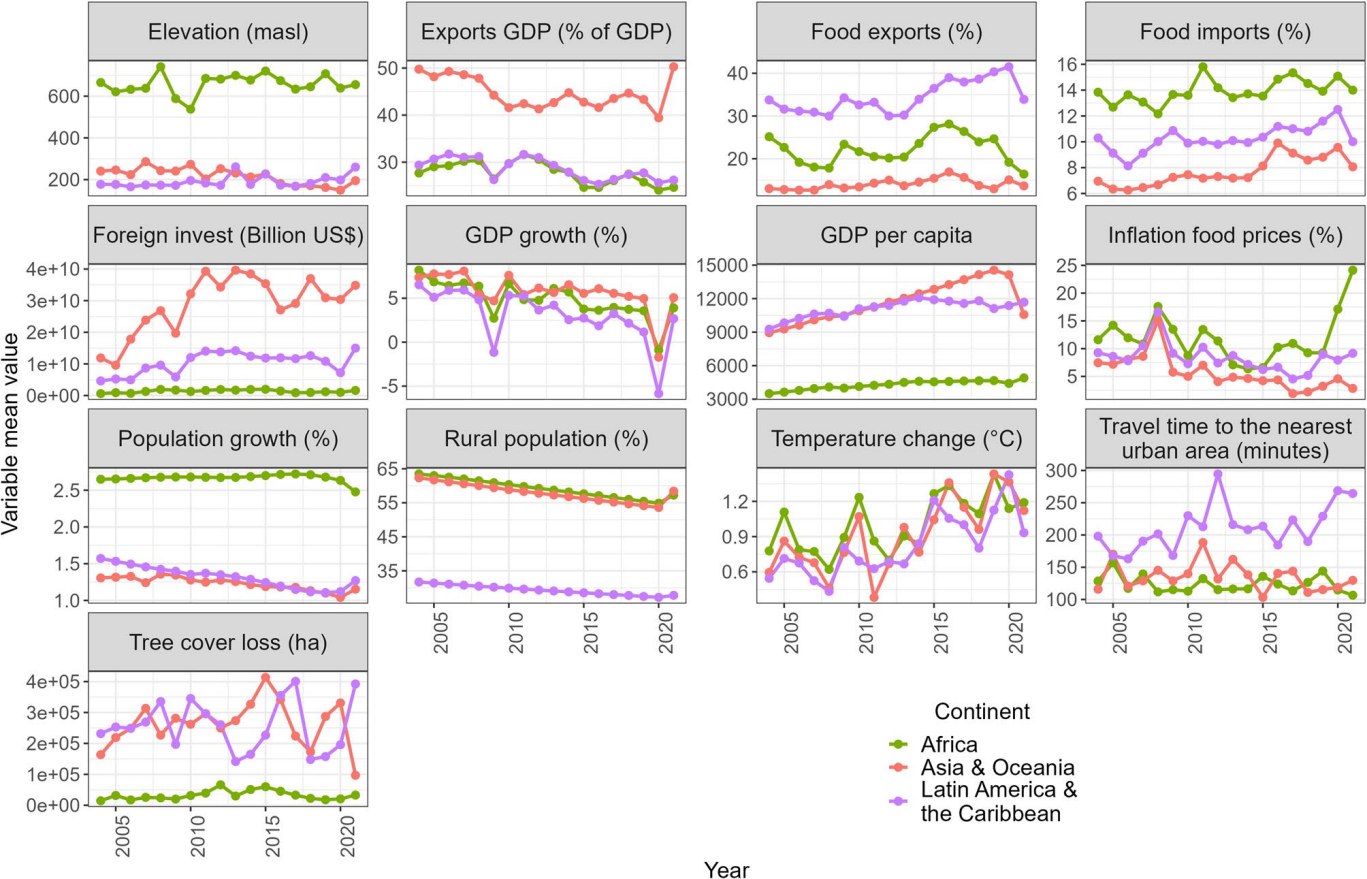
Mean and standard deviation of driver variables for each region. Consists of data for 40 countries for the period 2004–2021. Tree cover loss data are sourced from the Terra-i monitoring system. The elevation variable is a measure of the median elevation of pixels where tree cover loss was identified by Terra-i. ‘Foreign invest’ corresponds to cross-sectoral foreign direct investments. The travel time variable represents the average travel time from pixels where tree cover loss was identified to population centers with > 5000 inhabitants.

Twelve variables (Table 1; Fig. 1) consisting of data for 40 countries for the period 2004–2021 were included in global- and continental-scale XGBoost models.

**Table 1:** Indicators and corresponding datasets included in modeling to represent the different driver categories and food system dimensions. Tree cover loss pixels correspond to data from the Terra-i monitoring system. The data and metadata with links to the datasets used in the study are available online (see Data availability statement).

Food system dimension	Underlying drivers	Indicators	Variable name	Dataset	Source
Consumption/demand	Population and development changes	Population demographic transition	Population growth	Population growth (annual %)	World Bank
			Rural population	Rural population (% of total population)	World Bank
	Consumers’ income	Gross domestic product (GDP) growth	GDP growth	GDP growth (annual %)	World Bank
	Price changes	Commodity price changes	Food inflation	Food price inflation (%)	FAOSTAT
Production/supply	Poverty-related factors	GDP per capita	GDP per capita	GDP per capita based on purchasing power parity (current international $)	World Bank
	Access to infrastructure	Average travel time to population centers	Travel time	Average travel time from tree cover loss pixels to population centers with > 5000 people (minutes)	Own calculations based on Nelson et al.^40^
	Environmental factors	Climate change, climate shocks, extreme weather events	Temperature change	Temperature change (°C)	FAOSTAT
		Elevation	Elevation	Median elevation (MASL) of tree cover loss pixels	Own calculations based on Nelson et al.^40^
Trade/distribution	Market-related changes	Food imports	Food imports	% of merchandise imports	World Bank
		Food exports	Food exports	% of merchandise exports	World Bank
	Trade-related factors	Exports of goods and services	GDP exports	% of GDP from exports	World Bank
	Private and foreign public investments	Foreign direct investment	Foreign invest	Change over time in foreign direct investment (billion US$)	International Monetary Fund

Spearman’s rank-order correlations were used to assess the directional relationship between variables and tree cover loss (TCL). Correlations varied among scales and regions. However, three variables were found to have highly significant correlations (p < 0.001) with TCL at every scale and region assessed—FDI (positively correlated at each scale/region), percent of rural population (negatively correlated at each scale/region with a slightly weaker correlation in Africa [p < 0.01]), and GDP per capita (positively correlated across scales/regions except for Africa, which was negatively correlated at the p < 0.01 level) (Supplementary Figs. S3–S6). We considered both rural population and urban population growth variables for model inclusion. The two were found to have a perfect inverse correlation (ρ = -1), with rural population exhibiting a declining trend across regions (Fig. 1; Supplementary Figs. S7–S9). To avoid confounding results, only one of these variables was included in models; thus, rural population is used here as a proxy for population demographic changes associated with urbanization.

**Figure 2:**
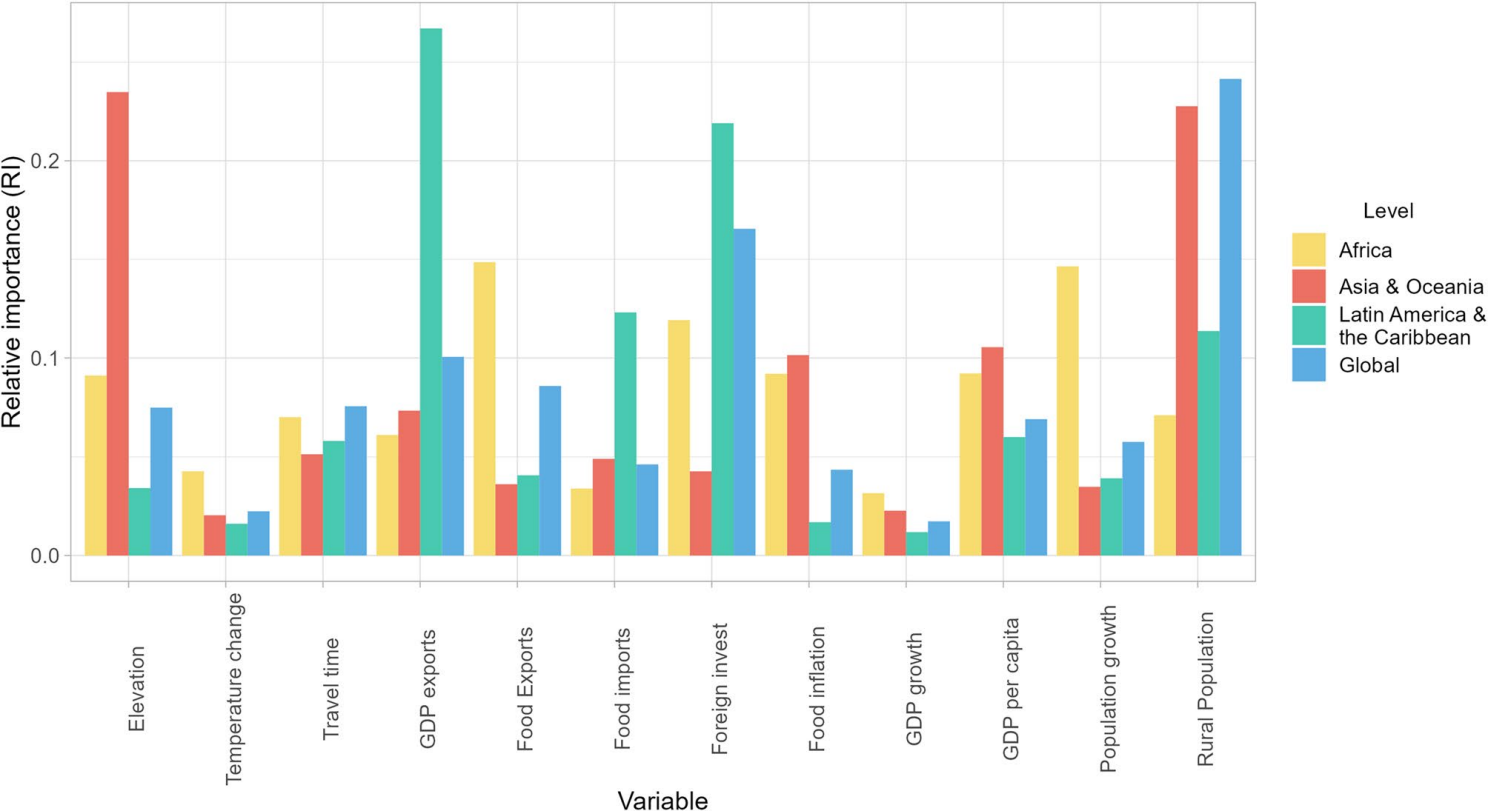
Relative importance of variables in explaining tree cover loss across scales. Consumption–demand factors include food inflation, GDP growth, population growth and percentage of rural population. Production–supply factors include GDP per capita, elevation (measure of median elevation of pixels where tree cover loss was identified), temperature change and travel time (average travel time from tree cover loss pixels to population centers with > 5000 people). Trade–distribution factors include GDP exports (% of GDP from exports), food exports, food imports and cross-sectoral foreign direct investments.

The global model explained a large amount of the variation in TCL observed in the 40 countries during the study period (R^2^ = 0.74). The two variables that contributed the most to explaining TCL at the global level were rural population from the consumption-demand dimension, explaining 24% of TCL variance in the model, and FDI from the trade-distribution dimension, explaining approximately 17% of observed TCL variance (Fig. 2). It should be noted that the influence of an individual country on regional model outputs could not be explored due to insufficient data observations for model execution.

The model for Asia and Oceania (n = 11 countries) explained 81% of TCL variance in the region. Elevation from the production-supply dimension and rural population from the consumption-demand dimension each explained approximately 23% of the variance in observed TCL, with both variables exhibiting significant negative correlations with TCL (Supplementary Fig. S5). Following these variables were GDP per capita and food inflation, explaining 11% and 10% of TCL in the model, respectively (Fig. 2).

For the Latin America and Caribbean (LAC) region (n = 12 countries), the variables in the model explained approximately 73% of observed TCL variance. A considerable amount of the region’s TCL was explained by two variables from the trade-distribution dimension. First, GDP of exports played a much larger role in explaining TCL in LAC than in other regions. This variable, which was negatively correlated with TCL (Supplementary Fig. S6), contributed to explaining 27% of TCL variance in the region. Second, FDI contributed to explaining 22% of the TCL variance in the model, while food imports (negatively correlated with TCL) explained 12% of TCL variance and rural population explained around 11% of TCL variance (Fig. 2).

Of the different scales and regions, the Africa-scale model (n = 17 countries) explained the least amount of TCL (R^2^ = 0.30). Food exports from the trade-distribution dimension and population growth from the consumption-demand dimension each explained approximately 15% of TCL variance. Correlations between these variables and TCL indicate that higher TCL corresponds to decreasing percentages of food exports and increasing percentages of population growth in the 2004–2021 period (Supplementary Fig. S4).

## Discussion

International efforts are making strides to combat deforestation while procuring food security, improved nutrition and poverty alleviation^41^. Current efforts are predominantly focusing on interventions within low opportunity cost production systems (e.g. shifting cultivation), agricultural intensification, zero-deforestation supply chain commitments centered on exports and connecting small-scale farmers to international markets^20,22,42,43^. More recently, calls for dietary changes have also gained prominence as a means of achieving sustainable food systems alongside land-based approaches^44,45^. However, there is a growing recognition that these strategies alone may not fully address the complex drivers of deforestation^20,46^. Deforestation for food production can be seen as the outcome of an unsustainable food system; and as such, interventions in the food system targeting its multiple dimensions are needed to achieve zero deforestation while feeding a growing global population. Through the modeling of food system drivers associated with its different dimensions, this study finds that consumption-demand dynamics, characterized by rural–urban population changes, and trade-distribution factors, specifically FDI, most explained global deforestation during 2004–2021.

Our findings align with growing evidence that increasing demand in urban areas is exerting significant pressures on forests^21,44^. Urbanization, often accompanied by rising incomes, leads to lifestyle changes marked by a notable rise in the consumption of (ultra)processed ‘convenience’ foods and animal-based products^26,27,35^. These changing diets increase demand for crops typically used in processed foods (e.g. palm oil, soy, cereals) and pasture and feed crops for livestock production^47,48^. This rise in demand can have a significant impact on prices, land values and, consequentially, production profitability, making regions with a comparative advantage in these crops and livestock products more attractive to FDI^49^. The proliferation of foreign investments, which surged following measures to reduce trade barriers via trade liberalization, has played a pivotal role in meeting escalating demands and, subsequently, facilitating increased consumption by influencing food production, supply and distribution dynamics^30,39^. For example, FDI has been instrumental in the proliferation of supermarkets and foreign-owned retail chains. These dynamics, in tandem with shifts in demand, have substantially contributed to the transformation of food systems in low- and middle-income countries, prominently exemplified by the "supermarketization" of food retail in these regions^15,29,30,50,51^.

Supermarket-based distribution of food can lower food prices through economies of scale, efficiency gains and coordination cost reductions^29,52^—a phenomenon that has been linked to inefficiency of the food system. Benton and Bailey^46^ argue that food production policies have predominantly centered on boosting agricultural productivity and promoting market liberalization to facilitate globalized trade. This has generated a surge in the supply of agricultural commodities, greater availability of calories and declining price trends. The prevalence of cheaper calorie options is now a cornerstone of (urban) diets, contributing to obesity and malnutrition, while global competition has provided incentives for producers who can maximize output while minimizing costs, often at the expense of the environment. This as well has underpinned the growth of the livestock sector with more affordable feed crops. These dynamics have consequentially shifted the burden of food production costs onto the environment, society and the healthcare system^46^.

Foreign investments have played a significant role in shaping both land and food systems. Several studies have illustrated how FDI in agriculture have influenced production systems and generated outcomes that are both positive, including improved productivity, food availability and access to water^53^, and negative, such as increased GHG emissions^54^, the expansion of monocultures and flex crops like soybean, oil palm, maize and sugarcane at the expense of biodiversity^33,55^, exacerbated inequality^56,57^ and, in some cases, reduced food security in the receiving country^58^. Several studies have also made clear connections between deforestation and large-scale land acquisitions, which are closely linked to FDI^59–63^. Lesser understood, however, is the broader influence of FDI beyond specific sectors in driving land use change and the mechanisms through which this is done, which warrants further investigation.

As such, our results point to the need to understand how FDI, irrespective of the sector, can indirectly influence land use decisions and drive deforestation outcomes. For instance, FDI can stimulate economic growth, infrastructure development, technology transfer and access to markets^64^, as well as increased rural–urban migration^53^, all of which can influence land use and land use change^4^. As our FDI variable encompasses all sectors, including those not directly linked to the food system, it is important to note that certain sectors, such as mining, logging and energy production, may have substantial monetary value compared to land investments, introducing complexities into the effects of FDI. The varying importance of FDI in our models could be influenced by these considerations. Nonetheless, this complexity underscores our limited understanding of how cross-sectoral investments affect the development dynamics that contribute to shaping food systems. Exploring the indirect effects of foreign investments on deforestation for food production remains challenging due to a lack of reliable and consistent data on and transparency in FDI flows and consequential land use changes over the long term, e.g.^61^. Nevertheless, the role of FDI in transforming food systems has been well documented, as briefly discussed above, and it is within this sphere that we gain some insights into how FDI’s influence on food systems may drive deforestation outcomes.

Findings from the correlations in LAC (Supplementary Fig. S6) indicate an inverse relationship between the percent of GDP from exports and tree cover loss. The strong explanatory power of this variable in the LAC model suggests that export-driven economies do not necessarily equate to high deforestation rates and that domestic consumption may be playing a larger role than export markets in driving deforestation. This is corroborated by Pendrill et al.^17^ who found that deforestation resulting from agricultural production was mostly for domestic demand, while 26% of the production leading to deforestation was exported for international demand. Lambin and Furumo^20^ also note a rise in deforestation associated with domestic markets and highlight that “for most commodities, major exporting firms are also active in domestic markets.” By leveraging their existing knowledge, resources and market power, these firms can further bolster a conducive environment for FDI, attracting investments aimed at enhancing competitiveness, market expansion and supply chain integration^65^. Supplementary Fig. S6 also denotes a negative correlation between FDI and GDP of exports, possibly reflecting investments toward sectors aiming to fulfill more of the local demand (e.g. transportation infrastructure, information and communication, renewable energy) rather than foreign demand^66^. Furthermore, the inverse relationship found for GDP of exports in LAC could also reflect the high occurrence of illegal land grabbing in the region, where small-scale production is often employed as a strategy to claim ownership of lands acquired through illegal means^67,68^.

The drivers considered in our Africa model offer only a partial understanding of deforestation trends in the region, emphasizing the need for additional variables to comprehensively capture these dynamics. However, data constraints for this region precluded the inclusion of additional variables in our models. Underlying drivers commonly identified in this region include weak governance^69,70^, little coordination or incoherence of policies and institutions^71–73^ and insecurity in land tenure^74,75^. Of the deforestation that could be explained by this analysis, trade-distribution and consumption-demand dynamics were the most influential. These results support previous studies arguing that production and supply-side dynamics could be playing a smaller or more localized role in driving deforestation than claimed by prior studies^76^ and that interventions in the demand- and trade-side of food systems are needed to achieve zero deforestation^14,44,77^. Moreover, the importance of exports in the Africa model aligns with recent research suggesting that there is a growing influence of distant markets on land use change in the African region^78–80^. Lastly, contrary to other regions, population growth emerged as a more influential driver than rural–urban transitions in Africa, likely because a greater proportion of the population is still rural^81^ and the region continues to experience unprecedented population growth^80^. DeFries et al.^21^ also observed a less pronounced association between deforestation and urban growth at the regional scale for Africa.

In Asia and Oceania, deforestation was most explained by drivers related to consumption-demand (particularly rural population decline) and production-supply (mainly elevation) dynamics, highlighting both food demand and production as major influential forces. The importance of rural–urban population changes in Asia and Oceania is underscored by the rapid urbanization trends this region has experienced in recent decades. Due to its large population, this region has the largest number of urban residents globally^81^. The elevation variable—a biophysical factor that influences land accessibility and suitability for agriculture—indicated that higher TCL was associated with lower elevations. The importance of elevation in Asia is likely related to the fact that it is more convenient to develop road infrastructure and agro-industrial crops such as oil palm in flat areas because they depend on machinery and require specific climate and soil conditions^82^. Oil palm, in particular, requires an elevation between 0 and 1500 m^83^. For example, substantial forest loss has been observed in the lowlands of Kalimantan (Southeast Asia) due to concessions for logging and oil palm cultivation^84,85^. The importance of elevation in this region could reflect the influence of global supply chains and respective demand for palm oil due to its use in food, beauty, chemical and fuel products^86^ on production systems in Asia. These findings suggest that further production-side interventions targeting production in low-elevation areas are needed to mitigate deforestation expansion and that demand-side interventions coupled with sustainable urban development strategies are needed to alleviate the strain on natural resources caused by the region’s rapid rates of urbanization^81^.

The availability of data continues to be a significant limitation for global studies. As a result, governance and institutional variables, such as rule of law, government effectiveness, control of corruption and political stability, could not be included in our models due to the degree of missing data observations across the study years for various countries^87^*.* However, Leblois et al.^16^ found that institutional quality variables were not significant drivers of forest loss. Conversely, studies have found that land tenure and land grabbing can influence deforestation^88^ and that this ties with foreign investment^89^ as well as with illegal economies^90^. Economic forces are underpinned by policies, institutions, governance and property rights/land tenure. While economic forces can drive the profitability of land use change, institutional, political and governance factors can either facilitate, exacerbate or alleviate the likelihood of forest conversion. Improved data completeness for these variables is critical for developing more holistic models that better capture these dynamics.

A further data limitation for this study is the inability to differentiate deforestation for production of food vs non-food commodities (e.g. for fuel and fiber). Strides were taken to ensure efforts focused on countries with the highest rates of deforestation according to the Global Forest Resources Assessment^1,91^ (see “Methods”), which considers deforestation to be the conversion of forest to other land use. This was done to reduce confusion between deforestation and other types of forest loss (e.g. forest fires, forest plantation logging). While non-food related production could not be excluded from the analysis, Gladek et al.^92^ estimate that the production of non-food crops and food crops for non-food uses account for 13% of global arable land.

Lastly, there is a need for improved data on and transparency in FDI flows. Understanding how FDI-driven deforestation is embedded within global supply chains and commodity networks requires further investigation. Mapping these dynamics can reveal the broader reach of FDI's influence. As well, improved data on land values^93^ is critically needed to better understand how FDI influences local land values and consequentially land use decisions. While research suggests that agriculture-driven deforestation has been predominately attributable to small-scale farming^94^, the activities of external actors and dynamics of foreign interests and how they influence the economic propellers of deforestation remain largely hidden.

While use of the best available data enabled us to incorporate drivers from the three dimensions of the food system, we recognize that additional drivers from the different dimensions, particularly on the production side, are needed to more fully model relationships between food system dynamics, land use change and deforestation. This will be contingent on future data improvements for such variables at different scales. Moreover, we acknowledge the importance of further research at national and local levels to reinforce our findings and enhance the robustness of our claims. By addressing these limitations and continuing to refine our models with improved data, we aim to provide more reliable insights into the drivers of deforestation and inform effective interventions.

## Conclusions and policy implications

While current discourse on deforestation recognizes the complex interlinkages between globalization, international trade of agricultural commodities and urbanization, particular emphasis has been placed on the role of consumer demand in distant markets in driving international trade and the function of global supply chains in linking deforestation to the consumption patterns of distant economies. Our results, however, suggest that foreign direct investments (FDI) contribute to explaining tree cover loss patterns nearly as much as urban consumer demand at the global level. Moreover, our results reflect the importance of changes in diets and food demand resulting from the interplay between demographic population transitions, international investments and trade as a major driver of deforestation outcomes.

Our study underscores the need to understand deforestation as an outcome of the complex interplay between the food system's multiple dimensions of consumption-demand, production-supply and trade-distribution dynamics. The interrelation of consumption-demand and trade-distribution dynamics as drivers of negative food system outcomes suggests that there is greater potential than what is currently recognized for synergies between improving food security and nutrition and reducing deforestation aside from local interventions in production systems. While production-based interventions hold a key role in improving food security and reducing GHG emissions associated with production practices and deforestation, coupling such approaches with trade and demand-side interventions has potential to achieve more holistic transformation of food systems and advances in climate action.

An analysis at the global scale inevitably sacrifices some degree of complexity related to finer scale deforestation dynamics. Nevertheless, our results are consistent with previous and emerging research indicating that demand- and trade-side dynamics are playing significant roles in driving deforestation at the global, Asia and LAC scales, suggesting that food system-based interventions could indeed be effective in reducing deforestation in these regions. Conversely, a notable deviation in the explanatory power of our food system model for Africa was observed, suggesting that variables outside the food system might play a larger role in driving deforestation in this region. Future in-depth studies should focus on the political, socioeconomic and environmental factors unique to Africa. Specifically, research should investigate the impacts of governance quality, land tenure security, poverty, infrastructure development and climate variability^80^ on deforestation patterns. Future studies can adapt the framework (Supplementary Fig. S1) and analytical approach presented here to examine national and subnational dynamics more closely, provided that sufficient data is available. It will be particularly interesting to analyze the driving forces associated with export-oriented production vs production for domestic consumption. This further will help identify entry points for interventions in food systems. Of interest as well is the potential to shift investment flows to achieve more sustainable outcomes.

The entanglement of food demand and consumption and foreign direct investments suggests that calls for dietary changes as a prominent solution to the unsustainability of food systems need to be supported with directions to reduce the negative externalities of public and private investments. Our findings indicate the imperative for targeted policy interventions to mitigate deforestation as an outcome of the food system. First, policies and regulations are needed to ensure FDI harmonize economic growth with ecological sustainability. Financial incentives, such as tax breaks or subsidies, could be used to incentivize FDI that lead to sustainable outcomes. Policy guidelines should incorporate ecological considerations alongside conventional economic factors when shaping strategies for attracting foreign investments in agriculture. Governments should also carefully evaluate both economic and ecological criteria when choosing foreign investors^54^. Second, a food systems approach should be incorporated in policies and regulations aiming to reduce deforestation^95,96^. Third, zero-deforestation regulations currently seen for international supply chains, such as the European Union Regulation on Deforestation-free Products, should be applied as well to domestic markets, requiring sustainable sourcing for domestically consumed products. Fourth, demand-side initiatives should be implemented alongside production and supply interventions for both domestic and export markets^20^. These could include public awareness campaigns, educational school programs and food labeling initiatives that aim to promote sustainable consumption. Lastly, as urban consumers become more disconnected from food sources, greater efforts are needed to increase transparency in and public awareness of commodity sources and distribution, production practices and associated environmental impacts.

## Methods

### Conceptual framework

Our conceptual framework for food system drivers of deforestation (Supplementary Fig. S1) was developed through a critical review and amalgamation of existing frameworks for underlying drivers of deforestation^4,97^ and drivers of food systems^24,25^. Our approach synthesized elements from the Geist and Lambin^97^ framework, tailoring it to the context of food consumption, production and trade. This involved reclassifying deforestation drivers according to their relevance to these three food system dimensions as outlined by Béné et al.^5^. We harmonized varying definitions of drivers, adopting a flexible interpretation to encompass a wider spectrum of potential influences. For a comprehensive integration of the two frameworks reflecting both land and food systems, institutional and policy factors not linked to food trade were retained in a supplementary fourth category. However, data limitations precluded the inclusion of variables from this category. Thus, the analysis focused on drivers from the three food system dimensions.

### Geographic scope and scale of analysis

The geographical scope of the study was limited to tropical and subtropical regions where the highest rates of deforestation occur. To concentrate efforts on areas of deforestation hotspots, we used data from the 2020 Global Forest Resources Assessment (FRA)^1^ and the complementary FRA Remote Sensing survey^91^ (information provided by the FAO for the period 2000–2018) to select the 40 countries with the highest rates of deforestation during 2000–2020. This was done by first selecting the countries that represented 85% of the global deforestation reported in the FRA database over the period 2010–2020. Second, this preliminary list was supplemented with countries from subregions where the FRA Remote Sensing Survey identified significant deforestation between 2000 and 2018. The second step was proposed to compensate for some shortcomings in the statistical data gathered in the FRA database (some countries having made projections of deforestation over long periods without measuring this indicator in a precise way).

The use of FRA data to select countries for the analysis allowed us to use spatial data that reflects tree cover loss while also aligning as best as possible with the FAO definition of deforestation (FAO defines deforestation as the “conversion of forest to other land use independently whether human-induced or not”^98^) and limiting confusion between deforestation and other types of forest loss (e.g. forest fires, forest plantation logging).

The countries consist of 17 countries in Africa, 9 countries in Asia, 12 in Latin America and the Caribbean (LAC) and 2 in Oceania (Supplementary Table S1). Data for these countries were aggregated at the global scale and at the scale of each continent with countries being the unit of analysis. Due to the number of observations for the two countries in Oceania (Australia and Papua New Guinea), analyses considered Asia and Oceania together.

### Data

Tree cover loss (TCL) data from the Terra-i monitoring system (www.terra-i.org) were used to analyze TCL trends in the selected 40 countries (Supplementary Fig. S2). The use of Terra-i is advantageous due to its vegetation cover change monitoring ability, its temporal resolution and its capacity for processing at the scale of several regions. Terra-i detects anthropogenic changes in vegetation cover at a pantropical scale in near real-time using satellite data and computational neural networks^99^. The system monitors variations in vegetation cover using the Normalized Difference Vegetation Index (NDVI) of the Medium Resolution Imaging Spectroradiometer (MODIS), providing a temporal resolution of 16 days and a spatial resolution of 250 m^100^. For this study, having a coarser spatial resolution compared to that of other forest cover change datasets, such as Hansen et al.^101^, was beneficial to reduce computational time and memory demands, given the global scale of the analysis and the inclusion of 40 countries. While this resolution may underestimate vegetation cover loss, it is capable of adequately capturing trends and patterns in vegetation cover change across regions^102^. This level of resolution was sufficient for our machine learning approach, as the analysis focused on identifying overarching patterns and trends throughout the study period. Terra-i provides up-to-date data from 2004 to the present year. Therefore, the study period was limited to 2004–2021, with data corresponding to cumulative pixels of TCL per year for the 40 countries.

We carried out an exhaustive search of variables and datasets for modeling drivers corresponding to our food system-centric framework. The inclusion of data for the study was determined using several selection criteria, considering data requirements for the selected analytical approach. First, variables needed to have time-series-cross-sectional data (i.e. spatial and temporal variation), and they needed to align with the objectives of the study. Therefore, variables that were related to direct drivers of deforestation or had no spatial or temporal variation (e.g. a single value across countries or years) were not considered. Second, variables needed to meet data availability requirements. Those that met the first criteria were included in exploratory assessments to determine the completeness of data sources. This consisted of calculating the percent of missing data observations for each variable across countries (n = 40) and years (from 2004 to 2021, n = 18) by summing the number of years with missing data in the study period for each country (n = 720 observations per variable). Variables that had considerable data gaps (missing ≥ 20% of observations) were excluded from the analysis. The threshold for the acceptable percentage of missing data observations was determined by considering the requirements for data imputation. Data imputation should not be applied to datasets missing 20% or more of observations as the estimates may not be reliable^103^.

This approach yielded 13 variables representing nine driver categories associated with consumption-demand, production-supply and trade-distribution dynamics of the food system. Two variables—rural population and urban population—were found to be perfectly correlated. To avoid confounding results, only one of these variables was included in models, resulting in a final database consisting of 12 variables (Table 1). Multiple imputation via the Amelia R package^104^ was used to fill the remaining data gaps in the final selection of datasets.

### Analysis

A machine learning approach utilizing Multiple Extreme Gradient Boosting (XGBoost) was used for modeling the time series data at the global and continental scale for Africa, Asia & Oceania and LAC. XGBoost is a gradient tree boosting algorithm and ensemble learning technique in which predictive performance is improved by combining the predictions from multiple models. This technique uses stepwise optimization of a tree ensemble to minimize the residuals of the current model^105,106^. This algorithm was selected for the analysis due to its proven efficiency in terms of time and memory when modeling complex non-linear relationships^105^. This algorithm also has been found to handle correlation among variables^107^.

Prior to constructing the models, we assessed correlations among all predictor variables using Spearman’s rank-order correlation. We then constructed a series of models for each region that contained different combinations of predictors that prevented including pairs of highly correlated variables. We found that the relationships among predictor variables maintained the same pattern with comparable R^2^ and relative importance values when all variables (except urban population, given its perfect correlation with rural population) were included in one model compared to when separate models were constructed for different correlated variables, supporting the notion that XGBoost is rather tolerable of correlation among predictors^107^. Therefore, we present results of models containing all predictor variables that satisfied model inclusion criteria. Furthermore, rather than selectively removing predictor variables with low explanatory power from models, we opted to retain all variables across models to better facilitate comparison of variable influence across regions and scales and a more comprehensive understanding of the relationships between predictors and the response variable (TCL). In this way, we chose to prioritize predictive performance over model simplicity.

For hyperparameter tuning, we employed a random search procedure to optimize key XGBoost hyperparameters, including learning rate (eta), maximum tree depth (max_depth), gamma, minimum child weight (min_child_weight), subsample ratio (subsample) and column sample by tree ratio (colsample_bytree). The final hyperparameter settings for all models are provided in Supplementary Table S2. For each geographical level (global, Africa, Asia & Oceania, LAC), 100 models with random combinations of these hyperparameters were executed, and the best-performing model was selected based on the Root Mean Square Error (RMSE) using a tenfold cross-validation procedure^108^. This process was repeated five times for each geographical level. The final model output for each region was the average of these five selected models to ensure robustness and minimize overfitting^109^. The outcome variable for the models is TCL, consisting of annual values of total TCL in hectares for the selected countries and years (2004–2021), calculated using data from the Terra-i monitoring system. Data processing and analysis were done with R software^110^.

### Supplementary Information

Supplementary Information.

## Data Availability

All data used in models and the corresponding metadata with links to the datasets used in the analysis are available online in the Harvard Dataverse (10.7910/DVN/6PS9WK).

## Code availability

R code used in this study is publicly available at 10.5281/zenodo.10529056. Code was run using R version 4.3.2.

## References

[1] FAO. *FRA 2020 Remote Sensing Survey*. (2022).

[2] Turner BL, Lambin EF, Reenberg A (2007). The emergence of land change science for global environmental change and sustainability. Proc. Natl. Acad. Sci..

[3] Meyfroidt P (2018). Middle-range theories of land system change. Glob. Environ. Change..

[4] Geist, H. J. & Lambin, E. F. *What Drives Tropical Deforestation? A Meta-Analysis of Proximate and Underlying Causes of Deforestation Based on Subnational Case Study Evidence* (2001).

[5] Béné C (2019). When food systems meet sustainability—Current narratives and implications for actions. World Dev..

[6] Stefanovic L, Freytag-Leyer B, Kahl J (2020). Food system outcomes: An overview and the contribution to food systems transformation. Front. Sustain. Food Syst..

[7] HLPE. *Nutrition and Food Systems. A Report by the High Level Panel of Experts on Food Security and Nutrition of the Committee on World Food Security* (2017).

[8] Ericksen PJ (2008). Conceptualizing food systems for global environmental change research. Glob. Environ. Change..

[9] Bortoletti, M. & Lomax, J. *Collaborative Framework for Food Systems Transformation. A Multi-Stakeholder Pathway for Sustainable Food Systems*. http://www.oneplanetnetwork.org/initiative/setting-table-our-children-improving-governance-food-systems (2019).

[10] Friis, C. Telecoupling: A new framework for researching land-use change in a globalised world. In *Telecoupling: Exploring Land-Use Change in a Globalised World* (eds. Friis, C. & Nielsen, J. Ø.) 49–67 (Palgrave Studies in Natural Resource Management, 2019). 10.1007/978-3-030-11105-2_3.

[11] Liu, J. *et al.* Framing sustainability in a telecoupled world. *Ecol. Soc.* **18**, (2013).

[12] Meyfroidt, P. Explanations in telecoupling research. In *Telecoupling* 69–86 (Springer International Publishing, 2019). 10.1007/978-3-030-11105-2_4.

[13] Eakin, H. *et al.* Significance of telecoupling for exploration of land-use change. In *Rethinking Global Land Use in an Urban Era*, vol. 14 (eds. Seto, K. C. & Reenberg, A.) 141–161 (MIT Press, 2014).

[14] Lambin EF, Meyfroidt P (2011). Global land use change, economic globalization, and the looming land scarcity. Proc. Natl. Acad. Sci. U. S. A..

[15] Garrett, R. & Rueda, X. Telecoupling and Consumption in Agri-Food Systems. In *Telecoupling: Exploring Land-Use Change in a Globalised World* (eds. Friis, C. & Nielsen J.) (Palgrave Studies in Natural Resource Management, 2019).

[16] Leblois A, Damette O, Wolfersberger J (2017). What has driven deforestation in developing countries since the 2000s? Evidence from new remote-sensing data. World Dev..

[17] Pendrill F (2019). Agricultural and forestry trade drives large share of tropical deforestation emissions. Glob. Environ. Change.

[18] Lambin EF (2018). The role of supply-chain initiatives in reducing deforestation. Nat. Clim. Change.

[19] Boucher D, Elias P (2013). From REDD to deforestation-free supply chains: The persistent problem of leakage and scale. Carbon Manag..

[20] Lambin EF, Furumo PR (2023). Deforestation-free commodity supply chains: Myth or reality?. Annu. Rev. Environ. Resour..

[21] Defries RS, Rudel T, Uriarte M, Hansen M (2010). Deforestation driven by urban population growth and agricultural trade in the twenty-first century. Nat. Geosci..

[22] Henders S, Ostwald M, Verendel V, Ibisch P (2018). Do national strategies under the UN biodiversity and climate conventions address agricultural commodity consumption as deforestation driver?. Land Use Policy.

[23] Jayathilake HM, Prescott GW, Carrasco LR, Rao M, Symes WS (2021). Drivers of deforestation and degradation for 28 tropical conservation landscapes. Ambio.

[24] Béné C (2019). Understanding food systems drivers: A critical review of the literature. Glob. Food Sec..

[25] Béné C (2020). Global drivers of food system (un) sustainability: A multi-country correlation analysis. PLoS One.

[26] Swinburn BA (2011). The global obesity pandemic: Shaped by global drivers and local environments. Lancet..

[27] Gómez MI, Ricketts KD (2013). Food value chain transformations in developing countries: Selected hypotheses on nutritional implications. Food Policy.

[28] Seto KC, Ramankutty N (2016). Hidden linkages between urbanization and food systems. Science..

[29] Reardon T, Timmer CP, Barrett CB, Berdegué J (2003). The rise of supermarkets in Africa, Asia, and Latin America. Am. J. Agric. Econ..

[30] Kearney J (2010). Food consumption trends and drivers. Philos. Trans. R. Soc. B Biol. Sci..

[31] McCullough, E. B., Pingali, P. L. & Stamoulis, K. G. Small farms and the transformation of food systems: An overview. In *The Transformation of Agri-Food Systems* 27–70 (Routledge, 2008).

[32] Khoury CK (2014). Increasing homogeneity in global food supplies and the implications for food security. Proc. Natl. Acad. Sci..

[33] Ceddia MG (2020). The super-rich and cropland expansion via direct investments in agriculture. Nat. Sustain..

[34] Drewnowski A, Popkin BM (1997). The nutrition transition: New trends in the global diet. Nutr. Rev..

[35] Herforth A, Ahmed S (2015). The food environment, its effects on dietary consumption, and potential for measurement within agriculture-nutrition interventions. Food Secur..

[36] IPES. *From Uniformity to Diversity: A Paradigm Shift from Industrial Agriculture to Diversified Agroecological Systems*. *International Panel of Ecperts on Sustainable Food Systems* (2016).

[37] Quinton JN, Govers G, Van Oost K, Bardgett RD (2010). The impact of agricultural soil erosion on biogeochemical cycling. Nat. Geosci..

[38] Amundson R (2015). Soil and human security in the 21st century. Science (1979).

[39] Thow AM, Hawkes C (2009). The implications of trade liberalization for diet and health: A case study from Central America. Glob. Health.

[40] Nelson A (2019). A suite of global accessibility indicators. Sci. Data.

[41] Bastos Lima MG, Kissinger G, Visseren-Hamakers IJ, Braña-Varela J, Gupta A (2017). The sustainable development goals and REDD+: Assessing institutional interactions and the pursuit of synergies. Int. Environ. Agreem..

[42] Garrett RD (2018). Intensification in agriculture-forest frontiers: Land use responses to development and conservation policies in Brazil. Glob. Environ. Change.

[43] Nepstad DC, Boyd W, Stickler CM, Bezerra T, Azevedo AA (2013). Responding to climate change and the global land crisis: REDD+, market transformation and low-emissions rural development. Philos. Trans. R. Soc. B Biol. Sci..

[44] Theurl MC (2020). Food systems in a zero-deforestation world: Dietary change is more important than intensification for climate targets in 2050. Sci. Total Environ..

[45] Vermeulen SJ, Park T, Khoury CK, Béné C (2020). Changing diets and the transformation of the global food system. Ann. N. Y. Acad. Sci..

[46] Benton TG, Bailey R (2019). The paradox of productivity: Agricultural productivity promotes food system inefficiency. Glob. Sustain..

[47] Zoomers A (2010). Globalisation and the foreignisation of space: Seven processes driving the current global land grab. J. Peasant Stud..

[48] Clark M (2020). The role of healthy diets in environmentally sustainable food systems. Food Nutr. Bull..

[49] Cotula L (2012). The international political economy of the global land rush: A critical appraisal of trends, scale, geography and drivers. J. Peasant Stud..

[50] Béné C (2022). Why the great food transformation may not happen—A deep-dive into our food systems’ political economy, controversies and politics of evidence. World Dev..

[51] Senauer, B. & Venturini, L. *The Globalization of Food Systems: A Conceptual Framework and Empirical Patterns*. (2005) 10.22004/ag.econ.14304

[52] Hawkes C (2005). The role of foreign direct investment in the nutrition transition. Public Health Nutr..

[53] Ben Slimane M, Huchet-Bourdon M, Zitouna H (2016). The role of sectoral FDI in promoting agricultural production and improving food security. Int. Econ..

[54] Kastratović R (2019). Impact of foreign direct investment on greenhouse gas emissions in agriculture of developing countries. Aust. J. Agric. Resour. Econ..

[55] Altieri MA, Nicholls CI, Henao A, Lana MA (2015). Agroecology and the design of climate change-resilient farming systems. Agron. Sustain. Dev..

[56] Basu P, Guariglia A (2007). Foreign direct investment, inequality, and growth. J. Macroecon..

[57] Reuveny R, Li Q (2003). Economic openness, democracy, and income inequality. Comp. Polit. Stud..

[58] Dogan B (2022). Does FDI in agriculture promote food security in developing countries? The role of land governance. Transnatl. Corp..

[59] Rulli MC (2019). Interdependencies and telecoupling of oil palm expansion at the expense of Indonesian rainforest. Renew. Sustain. Energy Rev..

[60] Conigliani C, Cuffaro N, D’Agostino G (2018). Large-scale land investments and forests in Africa. Land Use Policy.

[61] D’Odorico P, Rulli MC, Dell’Angelo J, Davis KF (2017). New frontiers of land and water commodification: socio-environmental controversies of large-scale land acquisitions. Land Degrad. Dev..

[62] Davis KF, Yu K, Rulli MC, Pichdara L, D’Odorico P (2015). Accelerated deforestation driven by large-scale land acquisitions in Cambodia. Nat. Geosci..

[63] Davis KF (2020). Tropical forest loss enhanced by large-scale land acquisitions. Nat. Geosci..

[64] te Velde, D. W. *Foreign Direct Investment and Development: An Historical Perspective*. (2006).

[65] Williamson PJ (2015). The competitive advantages of emerging market multinationals: A re-assessment. Crit. Perspect. Int. Bus..

[66] UNCTAD. *World Investment Report 2022: Regional Trends Latin America and the Caribbean*. (2022).

[67] Brito B, Barreto P, Brandão A, Baima S, Gomes PH (2019). Stimulus for land grabbing and deforestation in the Brazilian Amazon. Environ. Res. Lett..

[68] Rodríguez-de-Francisco JC (2021). Post-conflict transition and REDD+ in Colombia: Challenges to reducing deforestation in the Amazon. For. Policy Econ..

[69] Hansen CP, Lund JF, Treue T (2009). Neither fast, nor easy: He prospect of reduced emissions from deforestation and degradation (REDD) in Ghana. Int. For. Rev..

[70] Röder A, Pröpper M, Stellmes M, Schneibel A, Hill J (2015). Assessing urban growth and rural land use transformations in a cross-border situation in Northern Namibia and Southern Angola. Land Use Policy.

[71] Carodenuto S (2015). A methodological framework for assessing agents, proximate drivers and underlying causes of deforestation: Field test results from Southern Cameroon. Forests.

[72] Waiswa D, Stern MJ, Prisley SP (2015). Drivers of deforestation in the Lake Victoria Crescent, Uganda. J. Sustain. For..

[73] Tegegne YT, Lindner M, Fobissie K, Kanninen M (2016). Evolution of drivers of deforestation and forest degradation in the Congo Basin forests: Exploring possible policy options to address forest loss. Land Use Policy.

[74] Gillet, P., Vermeulen, C., Feintrenie, L., Dessard, H. & Garcia, C. Drivers of deforestation in the congo basin tropical forest. A review. *Biotechnol. Agron. Soc. Environ*. **20** (2016).

[75] Wehkamp J, Aquino A, Fuss S, Reed EW (2015). Analyzing the perception of deforestation drivers by African policy makers in light of possible REDD+ policy responses. For. Policy Econ..

[76] Ravikumar A, Larson AM, Myers R, Trench T (2018). Inter-sectoral and multilevel coordination alone do not reduce deforestation and advance environmental justice: Why bold contestation works when collaboration fails. Environ. Plan. C Polit. Space.

[77] Pendrill F (2022). Disentangling the numbers behind agriculture-driven tropical deforestation. Science (1979).

[78] Ordway EM, Asner GP, Lambin EF (2017). Deforestation risk due to commodity crop expansion in sub-Saharan Africa. Environ. Res. Lett..

[79] Berman, N., Couttenier, M., Leblois, A. & Soubeyran, R. Crop prices and deforestation in the tropics. (2021).

[80] Assede ESP (2023). Understanding drivers of land use and land cover change in Africa: A review. Curr. Landsc. Ecol. Rep..

[81] United Nations, Department of Economic and Social Affairs & Population Division. *World Urbanization Prospects: The 2018 Revision (ST/ESA/SER.A/420)* (United Nations, 2019).

[82] Rudel TK, Defries R, Asner GP, Laurance WF (2009). Changing drivers of deforestation and new opportunities for conservation. Conserv. Biol..

[83] Al-Sabaeei AM (2022). Utilization of palm oil and its by-products in bio-asphalt and bio-concrete mixtures: A review. Constr. Build Mater..

[84] Carlson KM (2013). Carbon emissions from forest conversion by Kalimantan oil palm plantations. Nat. Clim. Change.

[85] Curran LM (2004). Lowland forest loss in protected areas of Indonesian Borneo. Science (1979).

[86] Tapia JFD, Doliente SS, Samsatli S (2021). How much land is available for sustainable palm oil?. Land Use Policy.

[87] Wehkamp J, Koch N, Lübbers S, Fuss S (2018). Governance and deforestation—A meta-analysis in economics. Ecol. Econ..

[88] Kruid, S. *et al.* Beyond deforestation: Carbon emissions from land grabbing and forest degradation in the Brazilian Amazon. *Front. For. Glob. Change.* **4**, (2021).

[89] Wilkinson J, Reydon B, Di Sabbato A (2012). Concentration and foreign ownership of land in Brazil in the context of global land grabbing. Can. J. Dev. Stud. Revue canadienne d’études du développement.

[90] Castro-Nunez A, Mertz O, Buritica A, Sosa CC, Lee ST (2017). Land related grievances shape tropical forest-cover in areas affected by armed-conflict. Appl. Geogr..

[91] FAO. *Global Forest Resources Assessment 2020: Main Report* (2020).

[92] Gladek, E. *et al. The Global Food System: An Analysis* (2017).

[93] Coomes OT, Macdonald GK, De Waroux YLP (2018). Geospatial land price data: A public good for global change science and policy. Bioscience.

[94] Branthomme, A. *et al. How Much Do Large-Scale and Small-Scale Farming Contribute to Global Deforestation? Results from a Remote Sensing Pilot Approach* (FAO, 2023). 10.4060/cc5723en.

[95] Bhunnoo R (2019). The need for a food-systems approach to policy making. Lancet.

[96] Hawkes, C. *Brief I. Taking a Food Systems Approach to Policymaking: What, How, and Why*. https://knowledge4policy.ec.europa.eu/publication/taking-food-systems-approach-policymaking-what-how-why_en#:~:text=A%20food%20systems%20approach%20maximizes,reducing%20the%20risk%20of%20unintended (2022).

[97] Geist HJ, Lambin EF (2002). Proximate causes and underlying driving forces of tropical deforestation. Bioscience.

[98] FAO. *FRA 2020 Terms and Definitions* (2018).

[99] Reymondin, L. *et al. Terra-i: A Methodology for near Real-Time Monitoring of Habitat Change at Continental Scales Using MODIS-NDVI and TRMM*. http://www.terra-i.org/terra-i/publications.html (2012).

[100] Huete AR, Justice CO, van Leeuwen W (1999). MODIS vegetation index (MOD13). Algorithm Theor. Basis Doc..

[101] Hansen M (2013). High-resolution global maps of 21st-century forest cover change. Science.

[102] Céspedes J (2023). Has global deforestation accelerated due to the COVID-19 pandemic?. J. For. Res. (Harbin).

[103] Salgado, C. M., Azevedo, C., Proença, H. & Vieira, S. M. Missing data. In *Secondary Analysis of Electronic Health Records* 143–162 (2016) 10.1007/978-3-319-43742-2_13/FIGURES/12.

[104] Honaker J, King G, Blackwell M (2011). Amelia II: A program for missing data. J. Stat. Softw..

[105] Chen, T. & Guestrin, C. XGBoost: A Scalable tree boosting system. In *Proceedings of the 22nd ACM SIGKDD International Conference on Knowledge Discovery and Data Mining* 785–794 (ACM, 2016). 10.1145/2939672.2939785.

[106] Nishio M (2018). Computer-aided diagnosis of lung nodule using gradient tree boosting and Bayesian optimization. PLoS One.

[107] Elith J, Leathwick JR, Hastie T (2008). A working guide to boosted regression trees. J. Anim. Ecol..

[108] Kuhn, M. Futility analysis in the cross-validation of machine learning models. arXiv preprint arXiv:1405.6974 (2014).

[109] Ganzenmüller, R., Sylvester, J. M. & Castro-Nunez, A. What peace means for deforestation: An analysis of local deforestation dynamics in times of conflict and peace in Colombia. *Front. Environ. Sci.* **10**, (2022).

[110] R Core Team. *R: A Language and Environment for Statistical Computing* (2013).

