# Supplementary material for: Analysis of food system drivers of deforestation highlights foreign direct investments and urbanization as threats to tropical forests
Source: Sci Rep. 2024 Jul 16;14:15179. doi: 10.1038/s41598-024-65397-3 (PMC11252123; doi:10.1038/s41598-024-65397-3)
Supplement: Supplementary file 1 — Supplementary Information. [file 41598_2024_65397_MOESM1_ESM.pdf]

Analysis of food system drivers of deforestation highlights foreign direct investments and urbanization as threats to tropical forests

Janelle M. Sylvester[1,2], Diana María Gutiérrez-Zapata[1], Lisset Pérez-Marulanda[1], Martha Vanegas-Cubillos[1], Thilde Bech Bruun[2], Ole Mertz[2], Augusto Castro-Nunez[1*]

[1]International Center for Tropical Agriculture (CIAT), Km 17 Recta Cali-Palmira, Cali, Colombia
[2]Department of Geosciences and Natural Resource Management, University of Copenhagen, Copenhagen, Denmark
*augusto.castro@cgiar.org

Supplementary information

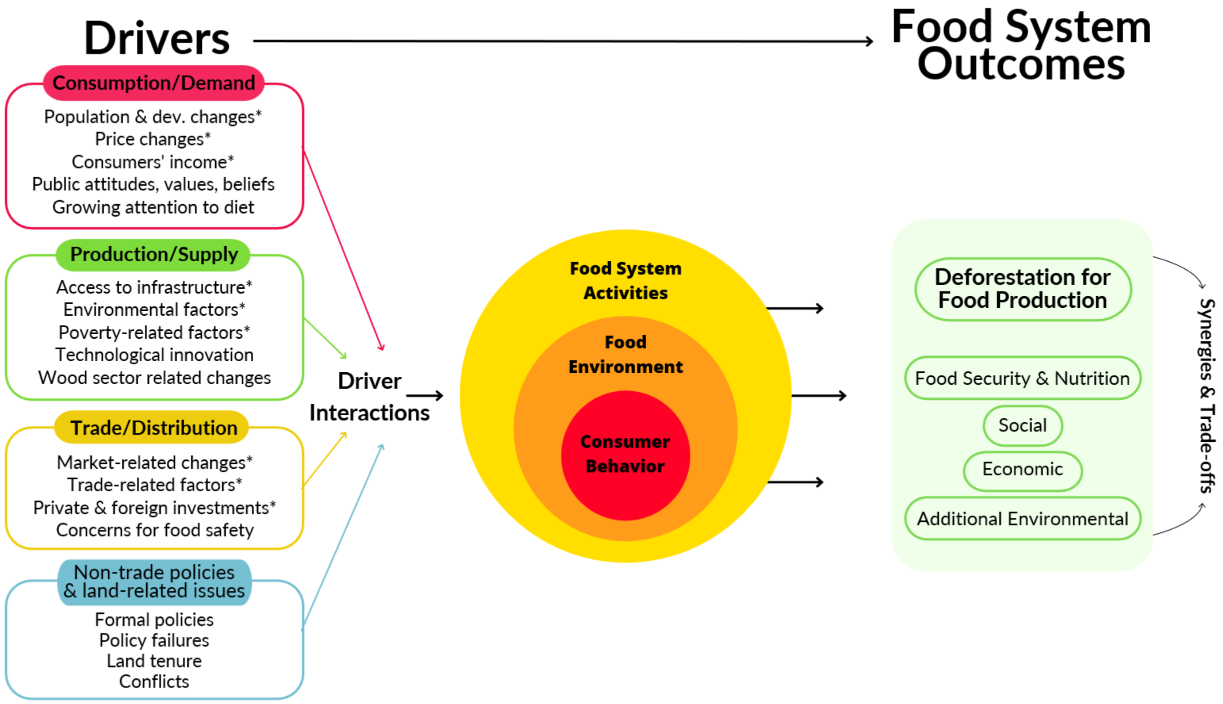

Figure S1. Conceptual framework for food system drivers of deforestation, integrating elements from Béné et al.[24] and Geist and Lambin[4]. The diagram illustrates how underlying drivers of deforestation[4] could be considered from a food system perspective and how they could interact with drivers of the food system[24] to shape deforestation outcomes.

The three dimensions of the food system are shown on the left with their associated drivers. Drivers with an asterisk (*) indicate those represented in models, which depended on data availability for the different proxies. A fourth category (Non-trade policies & land-related issues) is shown to account for drivers of land systems[4] that can interact with food system dynamics to drive deforestation for food production.

The three concentric circles form a simplified representation of the food system. The outermost circle, "Food System Activities," encapsulates food system activities and actors related to the production, processing, distribution, preparation, consumption and disposal of food[7]. The middle circle, "Food Environment," encompasses the “physical, economic, political and socio-cultural context in which consumers engage with the food system to acquire, prepare and consume food”[7]. The innermost circle, “Consumer Behavior,” represents “the choices made by consumers, at household or individual levels, on what food to acquire, store, prepare and eat, and on the allocation of food within the household (including gender repartition, feeding of children)”[7]. This nested structure implies that food system activities and elements frame the conditions of the food environment, which, in turn, shapes consumer choices. The different types of food system outcomes are shown on the right. The diagram was adapted from Béné et al.[24].

Table S1. Countries included in the analysis.

Region	Subregion	Country	Country code
Africa	Eastern and Southern Africa	Angola	AGO
Africa	Eastern and Southern Africa	Botswana	BWA
Africa	Western and Central Africa	Cameroon	CMR
Africa	Western and Central Africa	Chad	TCD
Africa	Western and Central Africa	Côte d'Ivoire	CIV
Africa	Western and Central Africa	Democratic Republic of the Congo	COD
Africa	Eastern and Southern Africa	Ethiopia	ETH
Africa	Western and Central Africa	Ghana	GHA
Africa	Western and Central Africa	Guinea	GIN
Africa	Eastern and Southern Africa	Madagascar	MDG
Africa	Eastern and Southern Africa	Mozambique	MOZ
Africa	Western and Central Africa	Nigeria	NGA
Africa	Eastern and Southern Africa	South Africa	ZAF
Africa	Northern Africa	Sudan	SDN
Africa	Eastern and Southern Africa	Uganda	UGA
Africa	Eastern and Southern Africa	United Republic of Tanzania	TZA
Africa	Eastern and Southern Africa	Zambia	ZMB
Asia	South and Southeast Asia	Cambodia	KHM
Asia	East Asia	China	CHN
Asia	South and Southeast Asia	India	IND
Asia	South and Southeast Asia	Indonesia	IDN
Asia	South and Southeast Asia	Lao PDR	LAO
Asia	South and Southeast Asia	Malaysia	MYS
Asia	South and Southeast Asia	Myanmar	MMR
Asia	South and Southeast Asia	Thailand	THA
Asia	South and Southeast Asia	Viet Nam	VNM
South America	South America	Argentina	ARG
South America	South America	Bolivia (Plurinational State of)	BOL
South America	South America	Brazil	BRA
South America	South America	Colombia	COL
South America	South America	Ecuador	ECU
North and Central America	Central America	Guatemala	GTM

North and Central America	Central America	Honduras	HND
North and Central America	North America	Mexico	MEX
North and Central America	Central America	Nicaragua	NIC
South America	South America	Paraguay	PRY
South America	South America	Peru	PER
South America	South America	Venezuela (Bolivarian Republic of)	VEN
Oceania	Oceania	Australia	AUS
Oceania	Oceania	Papua New Guinea	PNG

Table S2. Final hyperparameter settings of all XGBoost models executed with random search procedure. Model indicates the geographic level (Asia = Asia & Oceania; LAC = Latin America and the Caribbean). Each run corresponds to the best-performing model selected from 100 models initialized with random parameter settings (with five runs per geographic level, which were averaged to yield final results). R^2 is the adjustment/performance measure. RMSE is the Root Mean Squared Error, a performance measure and selection criterion. The hyperparameters include eta (learning rate), max_depth (maximum tree depth), gamma (minimum loss reduction), minimum child weight, colsample_bytree (subsample ratio of columns), subsample (subsample ratio of training instances) and nrounds (number of boosting iterations).

Model	Run	R^2	RMSE	eta	max_ depth	gamma	min_child _weight	colsample _bytree	subsample	nrounds
**Global**	run1	0.79	171549	0.10	3	2.30	5	0.46	0.83	874
**Global**	run2	0.71	176526	0.21	3	0.82	12	0.42	0.88	373
**Global**	run3	0.74	193704	0.07	7	8.15	11	0.46	0.41	981
**Global**	run4	0.74	201517	0.03	10	2.92	0	0.56	0.69	920
**Global**	run5	0.73	190747	0.04	7	7.75	7	0.57	0.78	939
**Africa**	run1	0.32	36304	0.04	8	4.43	14	0.53	0.64	84
**Africa**	run2	0.37	38534	0.10	8	0.75	0	0.52	0.91	361
**Africa**	run3	0.30	39250	0.08	5	4.84	5	0.43	0.44	474
**Africa**	run4	0.26	41817	0.13	5	1.90	6	0.45	0.79	193
**Africa**	run5	0.22	42530	0.10	4	0.83	1	0.65	0.40	127
**Asia**	run1	0.76	207555	0.06	7	1.85	12	0.58	0.88	404
**Asia**	run2	0.78	197145	0.02	6	1.49	4	0.67	0.63	254
**Asia**	run3	0.83	190012	0.05	2	3.47	6	0.42	0.74	927
**Asia**	run4	0.86	126099	0.02	10	0.71	1	0.39	0.94	784
**Asia**	run5	0.82	174245	0.03	4	1.07	3	0.59	0.56	664
**LAC**	run1	0.76	264017	0.22	2	1.24	2	0.52	0.64	345
**LAC**	run2	0.79	255864	0.06	6	0.61	7	0.30	0.52	230
**LAC**	run3	0.60	266607	0.49	9	5.12	8	0.54	0.90	956
**LAC**	run4	0.74	264191	0.12	3	3.64	4	0.42	0.99	345
**LAC**	run5	0.75	286897	0.09	5	5.18	6	0.60	0.55	638

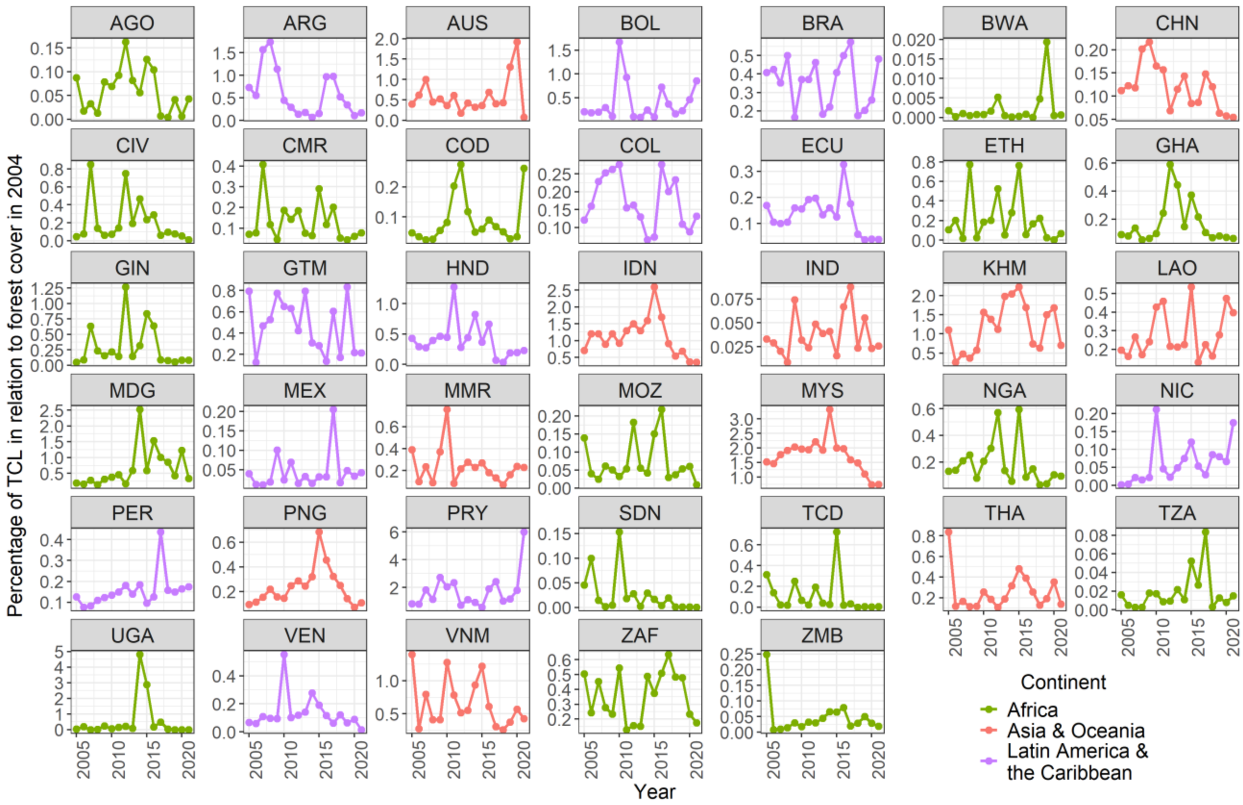

Figure S2. Percentage of gross tree cover loss (TCL) from Terra-i data between 2005 and 2021, relative to FAO-reported forest area in 2004 for countries included in the analysis (Source: https://data.worldbank.org/indicator/AG.LND.FRST.ZS?view=chart).

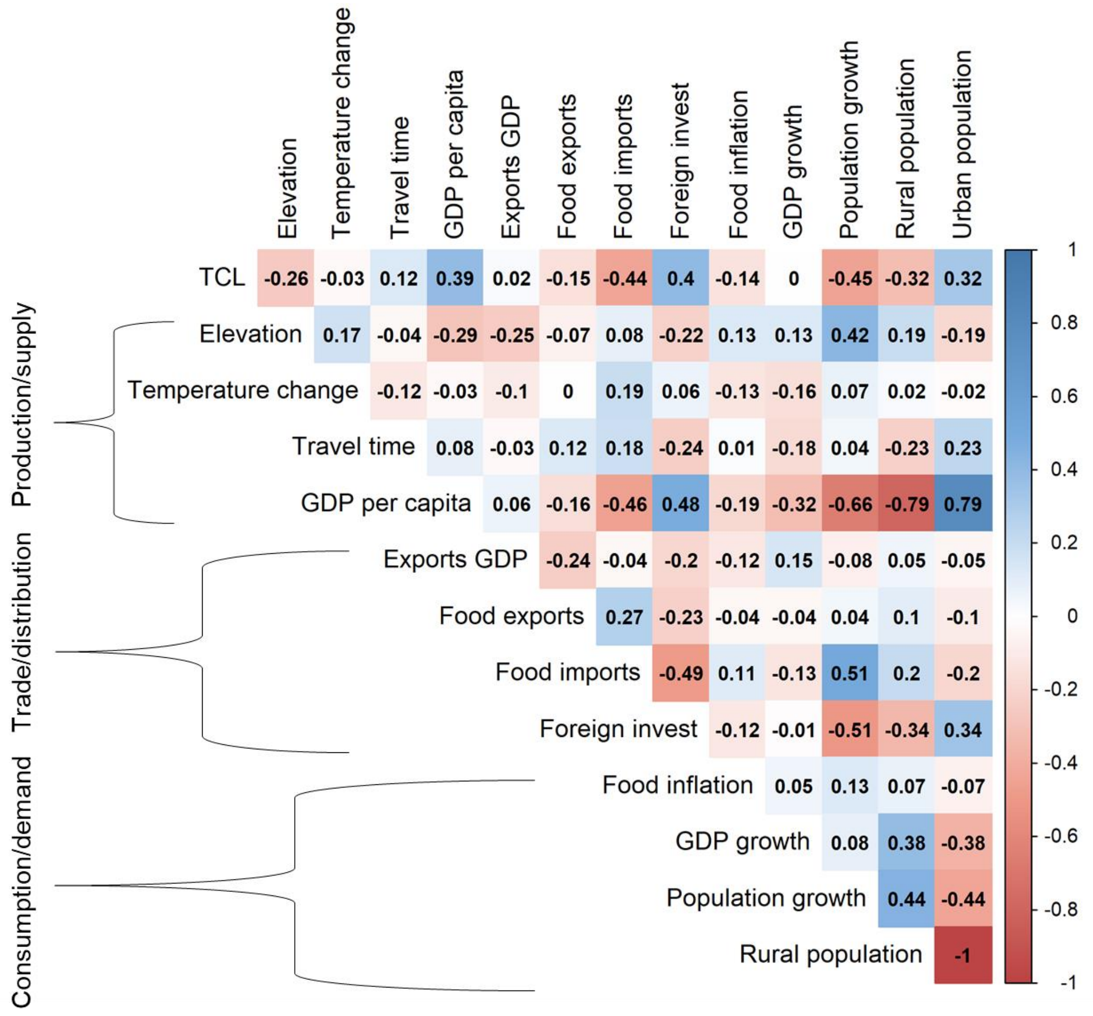

Figure S3. Spearman correlations in the time series database for production/supply, trade/distribution and consumption/demand variables at the global level. Blue cells represent a positive correlation while red represent a negative one. The larger the correlation, the darker the color. White cells mean non-significant correlations. The first row depicts the tested correlations for the response variable (tree cover loss). 'Foreign invest' corresponds to cross-sectoral foreign direct investments (FDI).

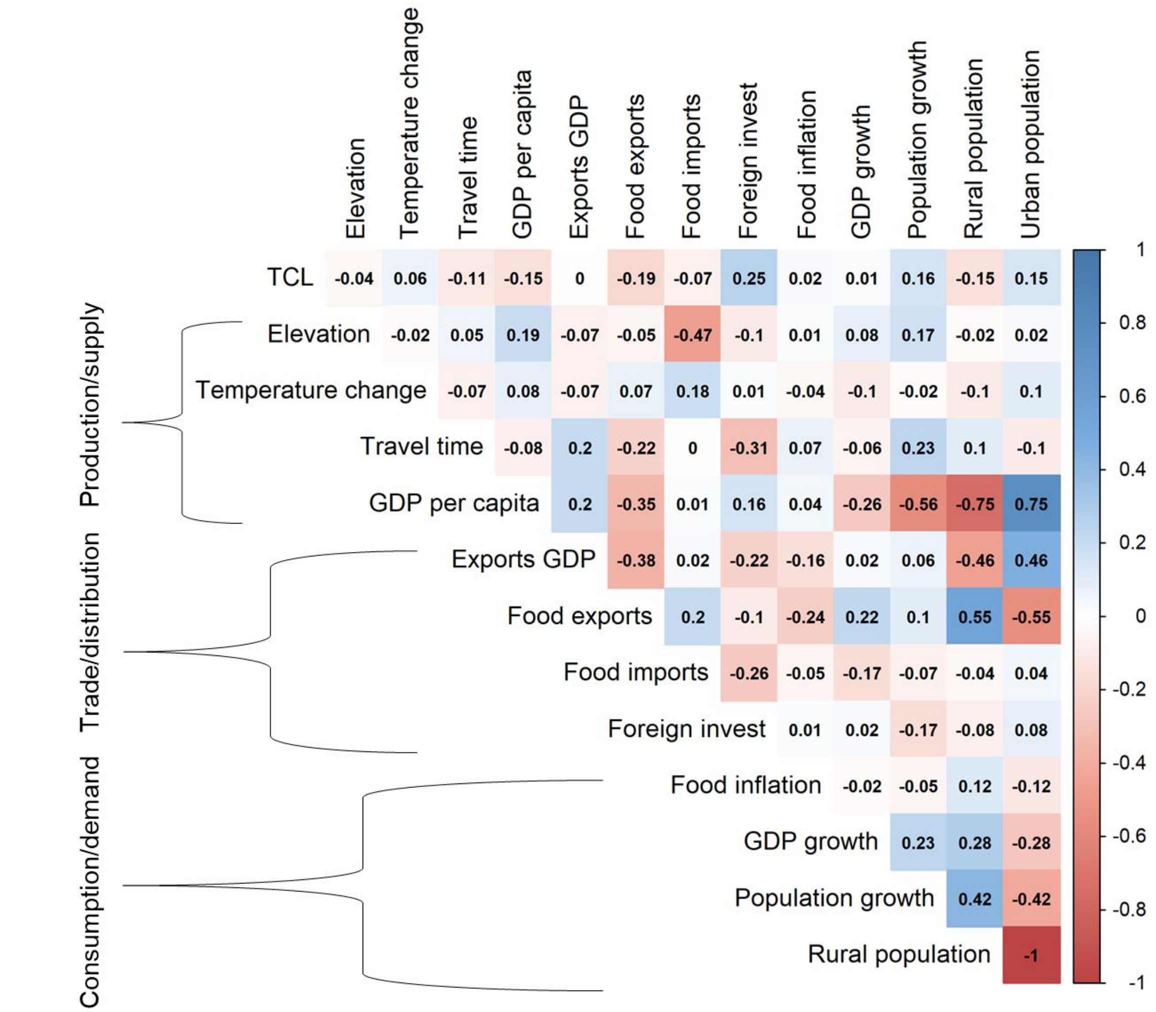

Figure S4. Spearman correlations in the time series database for production/supply, trade/distribution and consumption/demand variables at the Africa level. Blue cells represent a positive correlation, while red represent a negative one. The larger the correlation, the darker the color. White cells mean non-significant correlations. The first row depicts the tested correlations for the response variable (tree cover loss). 'Foreign invest' corresponds to cross-sectoral foreign direct investments (FDI).

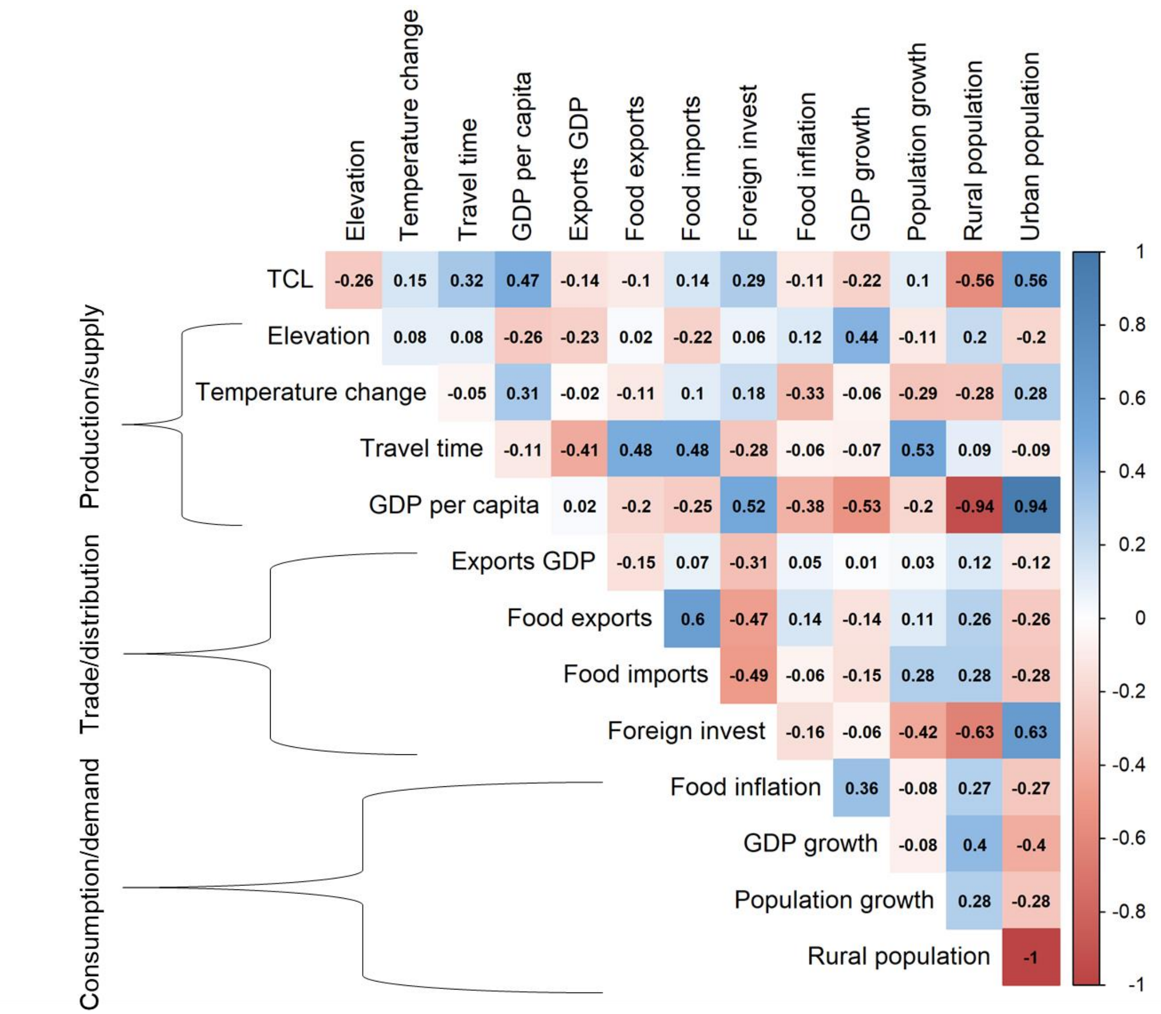

Figure S5. Spearman correlations in the time series database for production/supply, trade/distribution and consumption/demand variables at Asia & Oceania level. Blue cells represent a positive correlation, while red represent a negative one. The larger the correlation, the darker the color. White cells mean non-significant correlations. The first row depicts the tested correlations for the response variable (tree cover loss). 'Foreign invest' corresponds to cross-sectoral foreign direct investments (FDI).

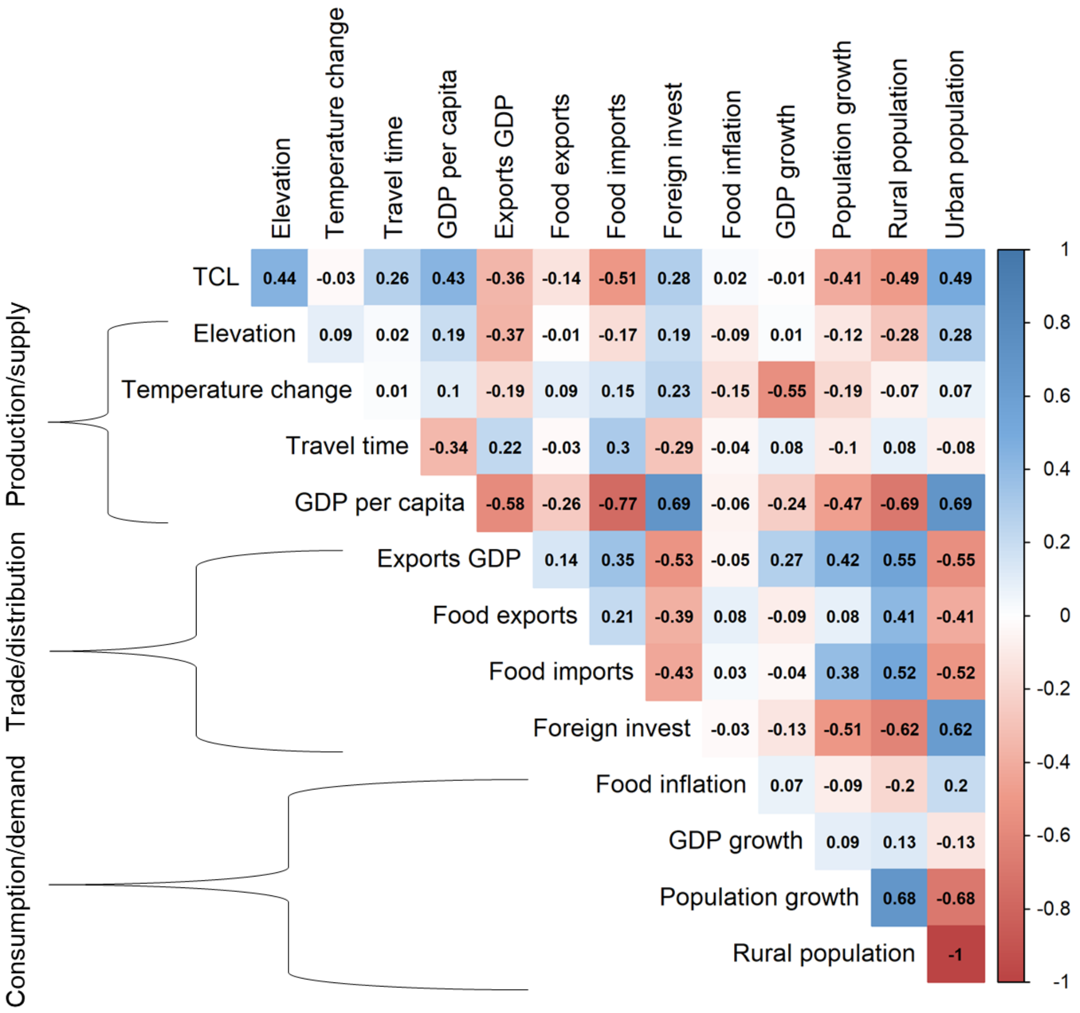

Figure S6. Spearman correlations in the time series database for production/supply, trade/distribution and consumption/demand variables at the Latin America and the Caribbean level. Blue cells represent a positive correlation, while red represent a negative one. The larger the correlation, the darker the color. White cells mean non-significant correlations. The first row depicts the tested correlations for the response variable (tree cover loss). 'Foreign invest' corresponds to cross-sectoral foreign direct investments (FDI).

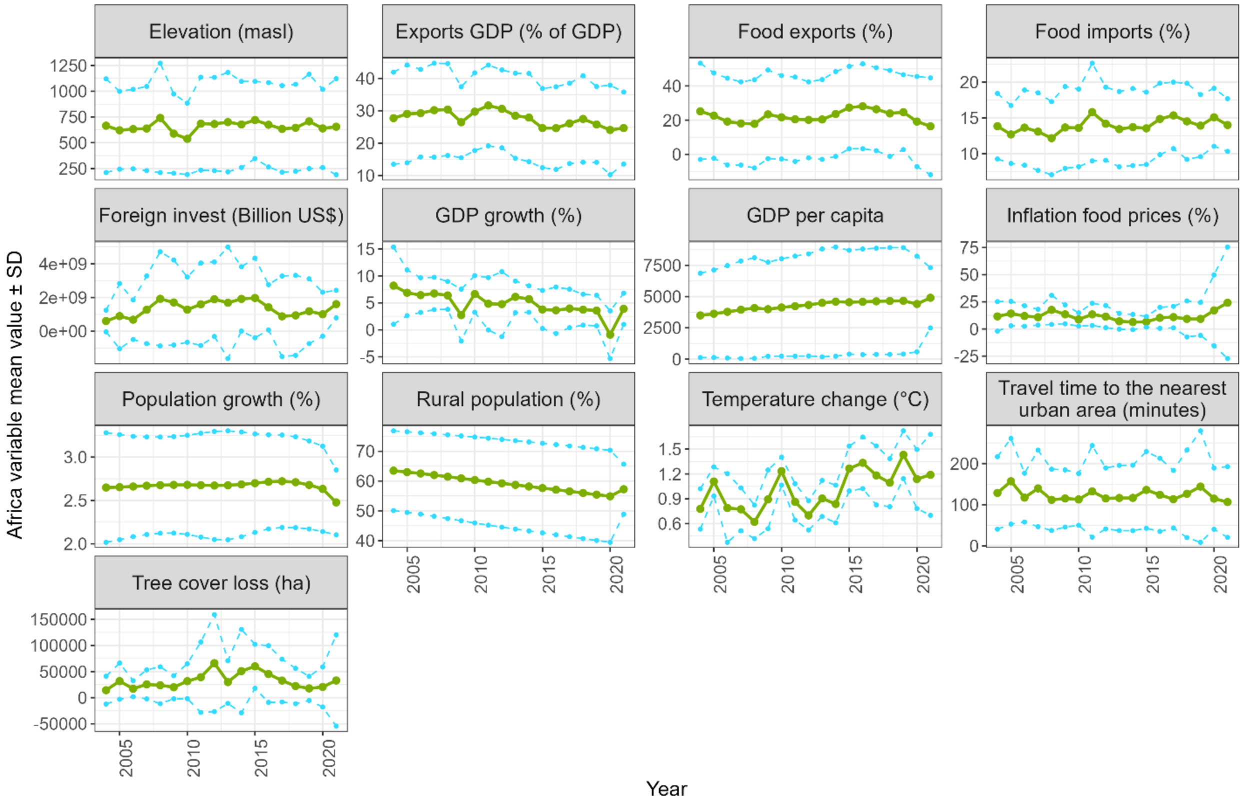

Figure S7. Mean and standard deviation of driver variables in Africa. Consists of data for 17 countries.

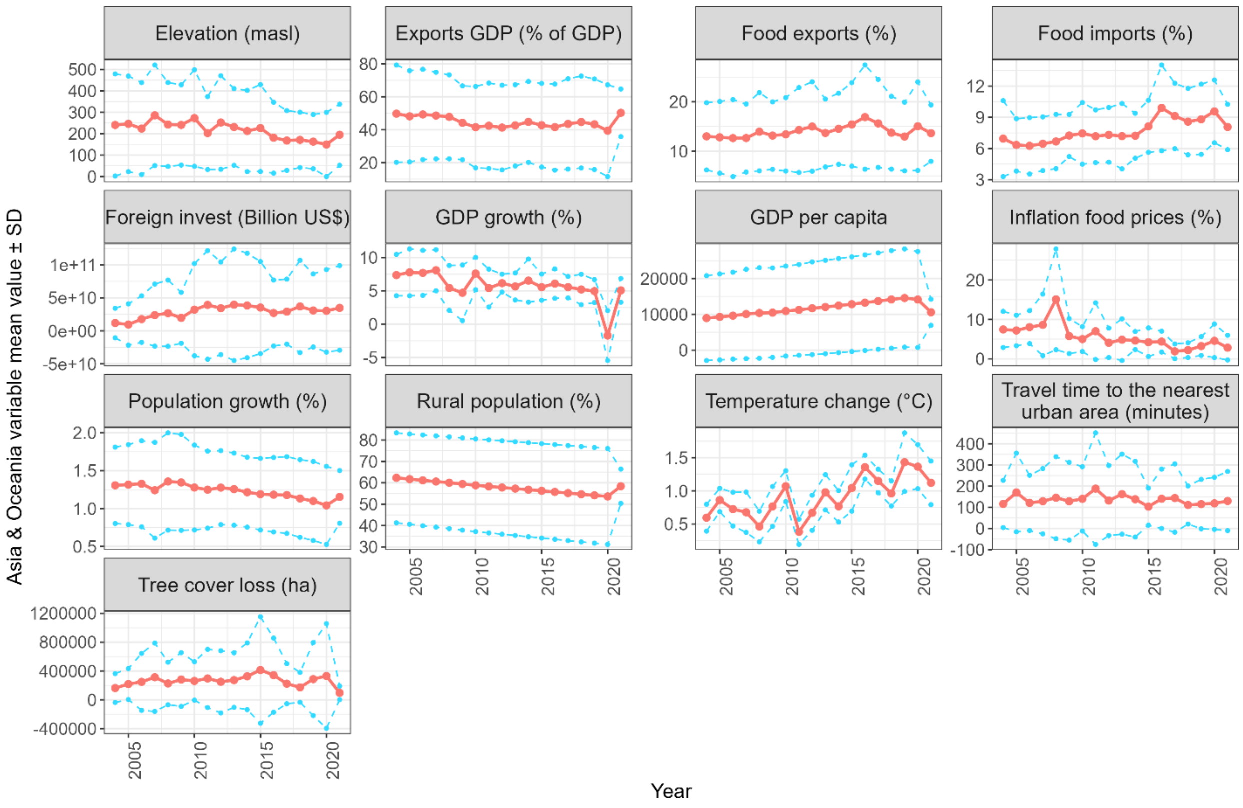

Figure S8. Mean and standard deviation of driver variables in Asia and Oceania. Consists of data for 9 countries in Asia and 2 in Oceania.

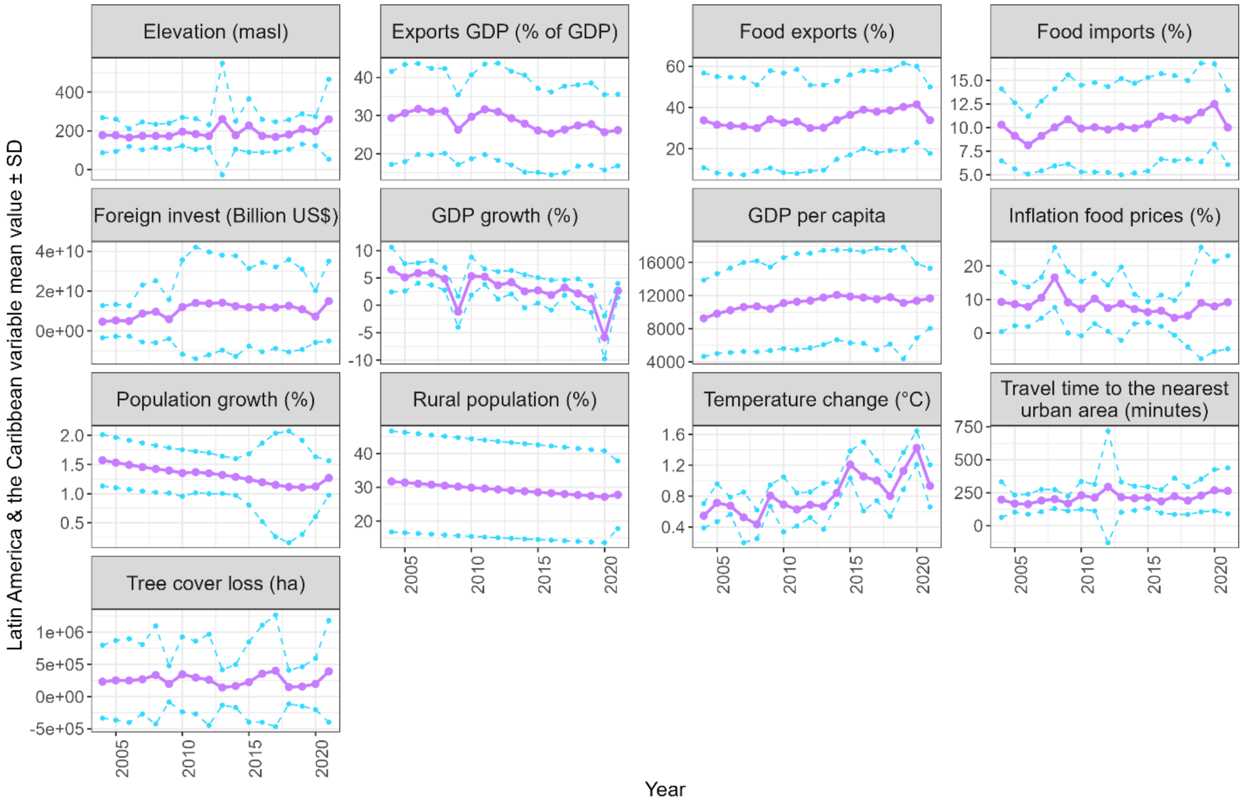

Figure S9. Mean and standard deviation of driver variables in Latin America and the Caribbean. Consists of data for 12 countries.
